# IgE‐reactivity profiles to allergen molecules in Russian children with and without symptoms of allergy revealed by micro‐array analysis

**DOI:** 10.1111/pai.13354

**Published:** 2020-10-04

**Authors:** Olga Elisyutina, Christian Lupinek, Elena Fedenko, Alla Litovkina, Evgenii Smolnikov, Nataliya Ilina, Dmitry Kudlay, Igor Shilovskiy, Rudolf Valenta, Musa Khaitov

**Affiliations:** ^1^ NRC Institute of Immunology FMBA of Russia Moscow Russia; ^2^ Division of Immunopathology Department of Pathophysiology and Allergy Research Centre for Pathophysiology, Infectiology and Immunology Medical University of Vienna Vienna Austria; ^3^ Sechenov First State Medical University Moscow Russia; ^4^ Karl Landsteiner University for Health Sciences Krems Austria

**Keywords:** allergen, allergy, Bet v 1, component‐resolved diagnosis, Fel d 1, food allergen molecules, food sensitization, micro‐array, PR10 protein, respiratory allergen molecules

## Abstract

**Background:**

The analysis of longitudinal birth cohorts with micro‐arrayed allergen molecules has provided interesting information about the evolution of IgE sensitization in children. However, so far no cross‐sectional study has been performed comparing IgE sensitization profiles in children with and without symptoms of allergy. Furthermore, no data are available regarding molecular IgE sensitization profiles in children from Russia.

**Methods:**

We recruited two groups of age‐ and gender‐matched children, one (Group 1: n = 103; 12.24 ± 2.23 years; male/female: 58/45) with symptoms and a second (Group 2: n = 97; 12.78 ± 2.23 years; male/female: 53/44), without symptoms of allergy according to international ISAAC questionnaire. Children were further studied regarding symptoms of allergy (rhinitis, asthma, atopic dermatitis) according to international guidelines, and skin prick testing with a panel of aeroallergen extracts was performed before sera were analyzed in an investigator‐blinded manner for IgE specific to more than 160 micro‐arrayed allergen molecules using ImmunoCAP ISAC technology.

**Results:**

IgE sensitization = or >0.3 ISU to at least one of the micro‐arrayed allergen molecules was found in 100% of the symptomatic children and in 36% of the asymptomatic children. Symptomatic and asymptomatic children showed a comparable IgE sensitization profile; however, frequencies of IgE sensitization and IgE levels to the individual allergen molecules were higher in the symptomatic children. Aeroallergen sensitization was dominated by sensitization to major birch pollen allergen, Bet v 1, and major cat allergen, Fel d 1. Food allergen sensitization was due to cross‐sensitization to PR10 pollen and food allergens whereas genuine peanut sensitization was absent.

**Conclusion:**

This is the first study analyzing molecular IgE sensitization profiles to more than 160 allergen molecules in children with and without symptoms of allergy. It detects similar molecular IgE sensitization profiles in symptomatic and asymptomatic children and identifies Bet v 1 and Fel d 1 as the predominant respiratory allergen molecules and PR10 proteins as the major food allergens and absence of genuine peanut allergy in Moscow region (Russia).

**Abbreviations**
AD: atopic dermatitis
AR: allergic rhinitis
AS: allergic asthma
IgE: immunoglobulin E
ISAC: immuno‐solid‐phase allergen chip
ISU: ISAC standardized units
MeDALL: mechanisms of the development of allergy
n: natural
OAS: oral allergy syndrome
PR‐10 allergen: Pathogenesis‐related allergen
r: recombinant

**Key Message**

Our study is the first cross‐sectional study analyzing the molecular sensitization profiles to a large number of allergen molecules by micro‐array in age‐ and gender‐matched children.

## 1 INTRODUCTION

Molecular allergology utilizes allergen molecules for improving the diagnosis of allergy and serves as a basis for the generation of new molecular allergy vaccines.^1^, ^2^ It has started with the isolation of the first allergen‐encoding DNA sequences^3^, ^4^, ^5^ and the first demonstration of the potential usefulness of recombinant allergen molecules for allergy diagnosis approximately 30 years ago.^6^, ^7^ Today, molecular allergy diagnosis is considered an important part of routine allergy diagnosis.^8^, ^9^ A major step in molecular allergy diagnosis was the development of multiplex allergy tests which are based on chips containing a large and comprehensive panel of micro‐arrayed allergen molecules which allow testing for IgE reactivity to multiple allergen molecules with small amounts of serum or other body fluids.^10^ Due to the fact that allergen micro‐arrays require only small volumes of serum, they are ideally suited for the assessment of IgE sensitization profiles in children where blood sampling and in vivo provocation testing may be challenging.^11^ Several studies have demonstrated the usefulness of micro‐array‐based determination of IgE sensitization profiles in children of which a few may be mentioned. For example, the evolution of the IgE reactivity profiles to individual grass pollen allergen molecules has been studied in birth cohorts providing important information regarding the development of IgE sensitization profiles in early childhood.^12^, ^13^ Using recombinant food allergen molecules, it has become possible to identify risk allergen molecules which may predict severity of food allergy reactions in children.^14^, ^15^ The deciphering of molecular IgE sensitization profiles using micro‐arrayed allergen molecules has been shown to be valuable for the personalized management of children suffering from multiple sensitizations^16^, ^17^ and for refining the prescription of allergen‐specific immunotherapy, allergen avoidance or diet.^18^, ^19^ Furthermore, molecular diagnosis seems to be useful for the assessment of respiratory allergies such as rhinitis and asthma^20^, ^21^, ^22^ as well as for certain types of food allergy, for example, oral allergy syndrome which is caused by cross‐reactivity between sensitizing pollen allergens and food allergens.^22^, ^23^

Interestingly, birth cohort studies indicate that it may be possible to predict, if a yet asymptomatic child may develop symptomatic allergy later in life based on early IgE sensitization profiles to micro‐arrayed allergen molecules.^24^ The assessment of maternal IgG antibodies transmitted by pregnant women to their offspring and determination of the IgE sensitization in the children thereafter has provided evidence that maternal IgG may protect children against allergic sensitization after birth.^25^

However, micro‐arrayed allergen molecules have opened yet another important field in allergy research, the assessment of regional molecular IgE sensitization profiles in populations from different parts of the world revealing interesting peculiarities of sensitization profiles.^26^ For example, house dust mite sensitization is very common in certain European countries whereas it is rare in other European countries due to climate reasons.^24^, ^27^ The molecular profiling of IgE sensitization patterns is very important for the development of allergen‐specific treatments and prevention. Unfortunately, such data are not available for certain large parts of the world such as Asia with only one study indicating lack of clinically relevant pollen allergen sensitization in the tropical climate of the Philippines.^28^ No assessments of molecular IgE sensitization profiles have yet been published for children from other large areas of the world such as North America, Africa and Russia.

Our study is the first detailed analysis of molecular IgE sensitization profiles in a cohort of Russian children from the Moscow region using more than 160 micro‐arrayed allergen molecules. Another unique aspect of this study is that we have enrolled two groups of age‐ and gender‐matched children, one group of children who according to the internationally accepted ISAAC questionnaire^29^ exhibited symptoms of allergy and one group in which children did not suffer from any symptoms of allergy. This study design allowed us to investigate the molecular IgE sensitization profiles in asymptomatic children and to compare them with those of symptomatic allergic children. Our results reveal a unique IgE sensitization profile of Russian children from the Moscow region to respiratory and food allergens, which is similar in symptomatic and asymptomatic children but differs in the two groups regarding frequencies and intensities (ie, IgE levels) of IgE recognition of the allergen molecules.

## 2 METHODS

### 2.1 Characterization of children with and without symptoms of allergy

In this study, 200 children attending the National Research Center—Institute of Immunology Federal Medical‐Biological Agency of Russia, Moscow, Russia, with their parents were enrolled with the goal to establish two equally sized, age‐ and gender‐matched groups of children. Permission from the local ethics committee and written informed consent from the parents were obtained. Then, a stepwise assessment was performed comprising:

#### 2.1.1 ISAAC questionnaire‐based assessment

**FIGURE 1:**
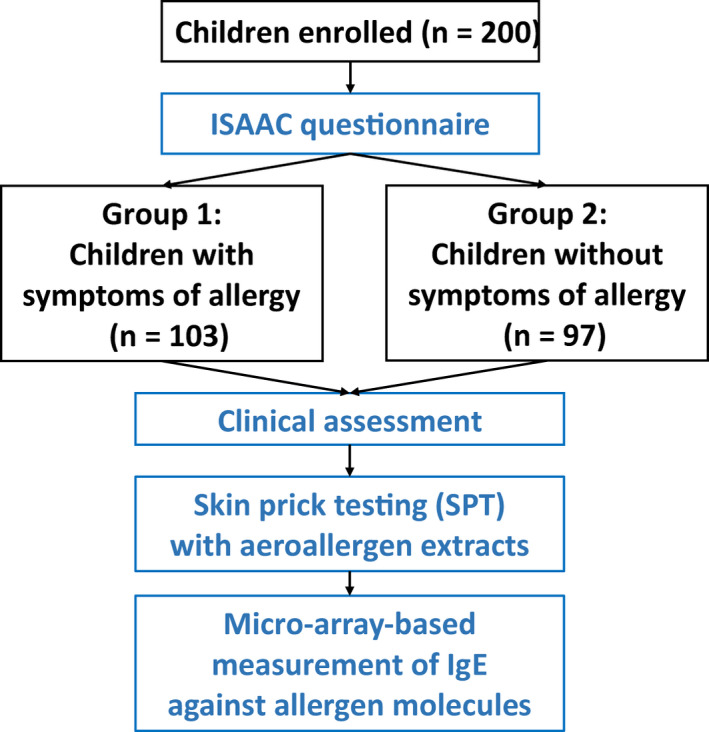
Scheme of the enrollment of children with and without symptoms of allergy, clinical characterization, and measurement of IgE specific for micro‐arrayed allergen molecules [Colour figure can be viewed at wileyonlinelibrary.com]

The parents were asked to fill out the “International Study of Asthma and Allergies in Childhood (ISAAC)” questionnaire,^29^ which is the most frequently internationally used questionnaire to establish if a subject suffers from symptoms of IgE‐associated allergy. The ISAAC questionnaire is available in multiple languages. We used an ISAAC version which had been translated into Russian language. Evidence for asthma was defined by a positive answer to the following questions, either “Has your child ever had wheezing or whistling in the chest at any time in the last 12 months?” or “In the last 12 months, has your child had a dry cough at night, apart from a cough associated with a cold or chest infection?” Allergic rhinitis was suspected after a positive answer to one of the following questions “Has your child ever had any of the following symptoms for at least one hour on most days (or on most days during the season if your symptoms are seasonal): watery, runny nose, sneezing (especially severe or even bouts of sneezing), nasal obstruction, nasal itching, conjunctivitis (red, itchy eyes), postnasal drip?” or “Have you ever had allergic rhinitis?” Questionnaire‐based evidence for atopic dermatitis required a positive answer to one of the following questions “Has your child ever had atopic dermatitis?” or “Has your child ever had an itchy rash that was coming and going for at least six months?” Based on the results from the ISAAC questionnaire, two groups of children were formed, one group (n = 103) with symptoms of allergy and a second group (n = 97) without symptoms of allergy (Figure 1).

#### 2.1.2 Clinical assessment

After the assignment of children in the symptomatic and asymptomatic group, a further clinical assessment of the children was performed, which included a detailed case history and a thorough physical examination (Figure 1). The clinical diagnosis of allergic rhinitis was based on recommendations by the European Academy of Allergy and Clinical Immunology^30^ and ARIA guidelines.^31^ The diagnosis of asthma was performed according to guidelines of the Global Initiative for Asthma/Global Strategy for Asthma Management and Prevention (available from: www.ginasthma.com).^32^ Atopic dermatitis was diagnosed based on international guidelines.^33^ In addition to ISAAC questionnaire‐based assessment, food allergy was assessed by an additional questionnaire checking symptoms of oral allergy syndrome (eg, pruritus of the lips, tongue, oral mucosa, burning sensations of the tongue, swelling of the lips or the tongue, swelling of the oral mucosa, laryngeal swelling, inflammation of the tongue or of the oral mucosa, perioral skin symptoms) urticaria, wheezing, dyspnea, nausea/vomiting, and gastrointestinal disorders that are associated with ingestion of common food allergen sources. The diagnosis of birch pollen–related oral allergy syndrome was based on a validated questionnaire approach.^34^ This questionnaire included questions regarding pruritus of the lips, tongue, oral mucosa, burning sensations of the tongue, swelling of the lips or the tongue, swelling of the oral mucosa, laryngeal swelling, inflammation of the tongue or of the oral mucosa, perioral skin symptoms, wheezing, dyspnea, nausea/vomiting, and gastrointestinal disorders which were associated with ingestion of apple, peach, carrot, nuts, or other fruits and vegetables. The questionnaire‐based assessment of OAS has been reported to have comparable diagnostic accuracy compared to oral provocation testing.^34^

#### 2.1.3 Skin prick testing

All children were then subjected to skin prick testing according to current guidelines using a panel of allergen extracts from respiratory allergen sources (tree pollen mix, grass pollen mix, weed pollen mix, house dust mite mix, cat, and dog) (Microgen, Moscow, Russia) (Figure 1).^35^

### 2.2 Micro‐array‐based determination of IgE reactivity to more than 160 allergen molecules

MeDALL allergen chips which had been developed based on the ImmunoCAP ISAC technology (Thermofisher, Phadia, Uppsala, Sweden) as described were used for measurement of specific IgE in sera from both groups of children (Figure 1).^36^ Arrays were scanned using a LuxScan 10K Microarray Scanner (CapitalBio Technology, Beijing, China) and analyzed with the micro‐array evaluation software version 3.1.2. The specific IgE levels measured by the MeDALL chip correspond to specific IgE levels measured with ImmunoCAP^®^ ISAC 112 and were standardized with a reference serum pool with known levels of specific IgE obtained from Thermofisher which is used as a reference serum pool (standard) in ImmunoCAP^®^ ISAC 112. Results are given in ISU‐IgE (ISAC standardized units). According to previous studies, the threshold for positivity of allergen‐specific IgE was defined by an IgE level of = or > 0.3 ISU‐IgE. The MeDALL chip showed equal or even slightly superior sensitivity in direct comparison to ImmunoCAP due to low background signals and a sensitive, fluorescence‐based detection system.^36^ Intra‐assay variation was determined to be approximately 8% in the IgE range of 1‐130 ISU‐IgE.^36^

### 2.3 Statistical analysis

Results (IgE levels) are given in medians, absolute (n), or relative numbers (%), where appropriate. The level of allergen‐specific IgE for each positive allergen molecule was compared between groups using the nonparametric *U* test. A *P* value of ≤.05 was considered statistically significant. Data were analyzed by using IBM SPSS Statistics.

## 3 RESULTS

### 3.1 Demographic and clinical characterization of children with and without symptoms of allergy

Based on ISAAC questionnaire data, we recruited 103 children (Group 1:58 males, 45 females) with a median age of 12.24 years who reported symptoms of allergy and a second group comprising 97 children (53 males, 44 females) with a median age of 12.7 years without symptoms of allergy (Table 1). Thus, the symptomatic and asymptomatic group of children were well‐matched regarding age and gender distribution. According to ISAAC and further detailed clinical assessment, rhinoconjunctivitis was by far the most common symptom among the 103 symptomatic children (n = 88) followed by atopic dermatitis (n = 55), asthma (n = 40), and food allergy (n = 35). Oral allergy syndrome (OAS) (n = 34) was by far the dominating symptom of food allergy followed by urticaria (n = 6) and anaphylaxis (n = 1) (Table 1). No symptoms of allergy were recorded in the group of 97 asymptomatic children. According to skin prick testing, sensitizations to the following allergen sources were found among the 103 symptomatic children (tree pollen: n = 69; cat: n = 64; dog: n = 43; grass pollen: n = 39; house dust mites (HDM): n = 39; weed pollen: n = 28). Among the 97 asymptomatic children, skin sensitivity was as follows: tree pollen: n = 12; HDM: n = 11; cat: n = 10; dog: n = 8; weed pollen: n = 4; and grass pollen: n = 2.

**TABLE 1:** Demographic and clinical characterization of children with and without symptoms of allergy from Moscow region, Russia (n = 200)

	Group 1 (patients with symptoms of allergy), n = 103		Group 2 (subjects without symptoms of allergy), n = 97	
Age, years M ± SD	12.24 ± 2.23		12.78 ± 2.23	
Gender (m/f)	58	45	53	44
ISAAC questionnaire data, n (%)				
Allergic rhinitis/conjunctivitis	88 (85.4)		0	
Asthma	40 (38.8)		0	
Atopic dermatitis	55 (53.4)		0	
Food allergy, in total	35 (33.9)		0	
Oral allergy syndrome	34 (33)		0	
Urticaria	6 (5.8)		0	
Anaphylaxis	1 (0.9)		0	
Skin prick tests with allergen extracts, positive results, n (%)				
Tree pollen (mix)	69 (66.9)		12 (12.3)	
Grass pollen (mix)	39 (37.8)		2 (2.1)	
Weed pollen (mix)	28 (27.1)		4 (4.1)	
House dust mite (mix)	39 (37.8)		11 (11.3)	
Cat	64 (62.1)		10 (10.3)	
Dog	43 (41.7)		8 (8.2)	

### 3.2 IgE sensitizations to major birch pollen allergen Bet v 1 and cat‐derived Fel d 1 dominate the hierarchy of aeroallergen sensitization

**FIGURE 2:**
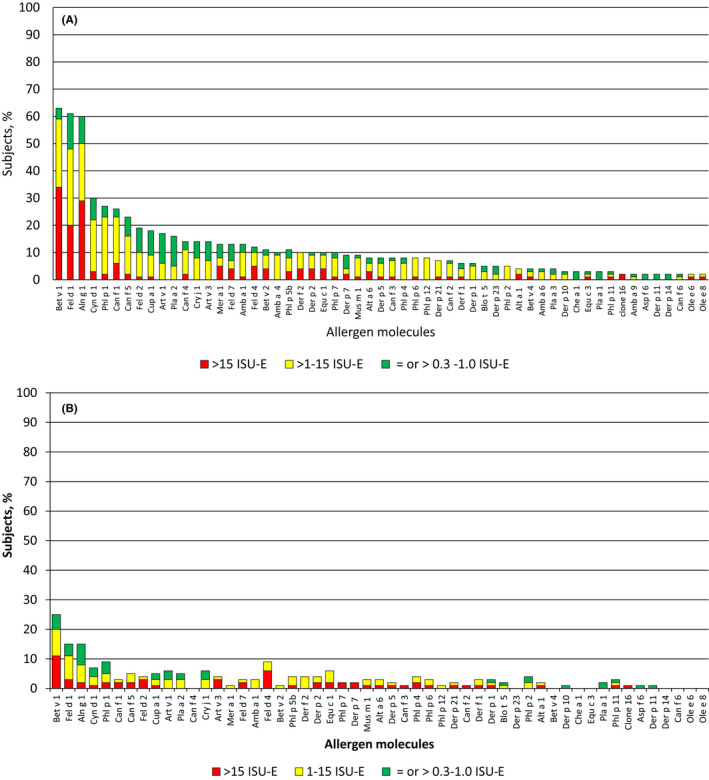
Hierarchy of the aeroallergen molecules according to frequencies of recognition (*y*‐axes: percentages of reactive children and IgE levels by color code) in children with (A) and children without symptoms of allergy (B) [Colour figure can be viewed at wileyonlinelibrary.com]

Sensitization to aeroallergens was dominated by two major allergen molecules, major birch pollen allergen Bet v 1 (Group 1:63.1%; group 2:25.7%) and the major cat allergen Fel d 1 (Group 1:61.1%; group 2:15.4%) (Figure 2A; Table S1). In the symptomatic children, the following allergen molecules which are indicative for certain allergen sources were next in the hierarchy: major timothy grass pollen allergen Phl p 1 (grass pollen) (27.1%), major dog allergen Can f 1 (dog) (26.2%), major cypress allergen Cup a 1 (cypress pollen) (18.4%), major mugwort pollen allergen Art v 1 (mugwort pollen) (16.5%), major plane tree pollen allergen Pla a 2 (plane tree pollen) (15.5%), major cedar pollen allergen Cry j 1 (cedar pollen) (13.5%), major ragweed allergen Amb a 1 (ragweed pollen) (12.6%), and major HDM allergen Der p 2 (HDM) (19.7%) (Figure 2A; Table S1). HDM sensitization was thus relatively rare in our population. Interestingly, sensitization to the mold Alternaria as indicated by the major allergen Alt a 1 was very rare (4/103) as was IgE sensitization to other mold allergens from Aspergillus and Cladosporium. No child was sensitized to ash pollen as indicated by IgE reactivity Ole e 1 which cross‐reacts with the major ash allergen Fra e 1.^37^ Likewise, no IgE sensitizations to cockroach, Parietaria, and Russian thistle (saltwort) were found among the symptomatic and asymptomatic children. Cross‐reactive plant allergens such as profilins (eg, Mer a 1, Bet v 2, Phl p 12) and two‐EF‐hand calcium‐binding allergens (polcalcins) (eg, Phl p 7, Bet v 4) were recognized by approximately 10% of the symptomatic children (Figure 2A; Table S1).

### 3.3 Children with symptoms of allergy show similar molecular IgE sensitization profiles to aeroallergens as children without symptoms of allergy: frequencies and IgE levels are different

The hierarchy of aeroallergen molecules recognized by asymptomatic children was very similar to that of symptomatic children (Figure 2B). Again major birch pollen allergen, Bet v 1 (25.7%), and major cat allergen Fel d 1 (15.4%) were the by far most frequently recognized allergens (Figure 2A,B; Table S1) but allergen‐specific IgE levels were significantly lower in asymptomatic children as compared to the symptomatic children (Table S1). The further hierarchy of IgE recognition was also similar to that of the symptomatic children Phl p 1 (9.2%) > Art v 1 (6.2%) = Cry j 1 (6.2%) > Cup a 1 (5.1%) = Pla a 2 (5.1%), and again allergen‐specific IgE levels were significantly lower than in the symptomatic children (Table S1). Thus, children without symptoms had similar IgE recognition profiles as compared to symptomatic children but specific IgE levels and sensitization rates were lower.

### 3.4 Food allergen sensitization is dominated by cross‐reactivity to PR10 allergens, whereas genuine peanut allergy is rare

**FIGURE 3:**
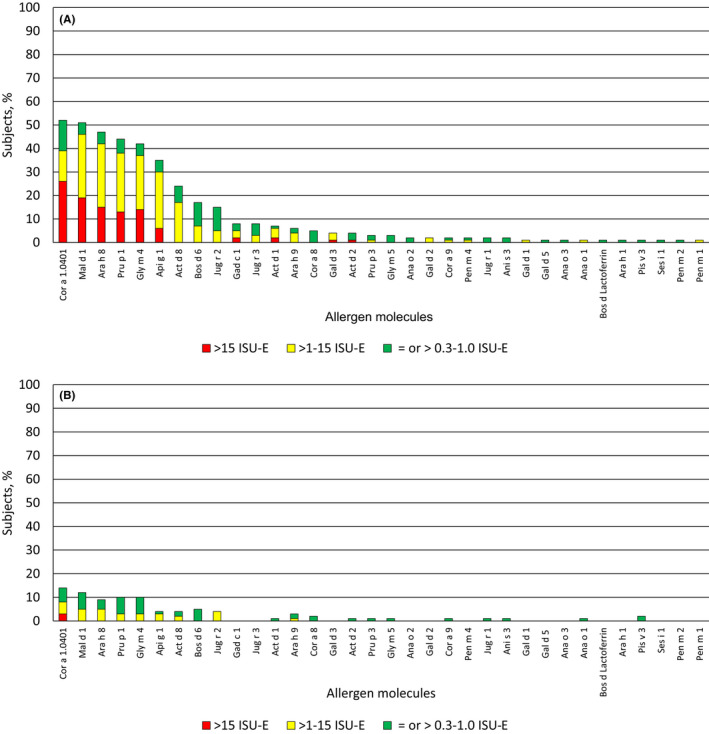
Hierarchy of the food allergen molecules according to frequencies of recognition (*y*‐axes: percentages of reactive children and IgE levels by color code) in children with (A) and children without symptoms of allergy (B) [Colour figure can be viewed at wileyonlinelibrary.com]

IgE sensitization to food allergens was dominated by PR10 proteins which cross‐react with the major birch pollen allergen Bet v 1.^38^ The major hazelnut allergen Cor a 1 (52.4%) and the major apple allergen Mal d 1 (51.4%) were the most frequently recognized PR10 allergens in the symptomatic children followed by Ara h 8 (peanut), Pru p 1 (peach), Gly m 4 (soybean), Api g 1 (celery), and Act d 8 (kiwi) (Table S2; Figure 3A). IgE sensitization to the PR10 allergens dominated also in the children without symptoms but fewer children were sensitized and specific IgE levels were significantly lower as in the symptomatic children (Table S2; Figure 3B). Interestingly, bovine serum albumin, Bos d 6, which is present in cow´s milk was the next most frequently allergen recognized by IgE from symptomatic (16.5%) and asymptomatic children (5.1%) (Table S2). However, we did not find any child in the two groups which showed IgE reactivity to the major cow´s milk allergens (caseins, lactalbumin, ß‐lactoglobulin) suggesting that IgE reactivity to Bos d 6 may result from IgE cross‐reactivity to other albumins such as cat albumin, Fel d 2 which was recognized frequently (ie, 19.4%) by symptomatic children (Table S1). Thus, Jug r 2 (14.5%) (walnut), Gad c 1 (7.8%) (fish), Act d 1 (6.8%) (kiwi), lipid transfer proteins (ie, peanut Ara h 9:5.8%; hazelnut Cor a 8:4.8%), and Gal d 3 (3.9%) (egg) were the next most frequently recognized food allergen molecules (Table S2; Figure 3A,B). We did not find any child with IgE reactivity to the genuine peanut allergens Ara h 2, Ara h 3, or Ara h 6. Only one symptomatic child had a very low Ara h 1‐specific IgE sensitization (Table S2). Likewise, none of the children had an IgE sensitization to the major cow´s milk allergens or to the wheat allergens (Table S2).

### 3.5 Pollen sensitization is dominated by birch pollen followed by grass pollen, cypress, mugwort, plane tree, cedar, and ragweed

**FIGURE 4:**
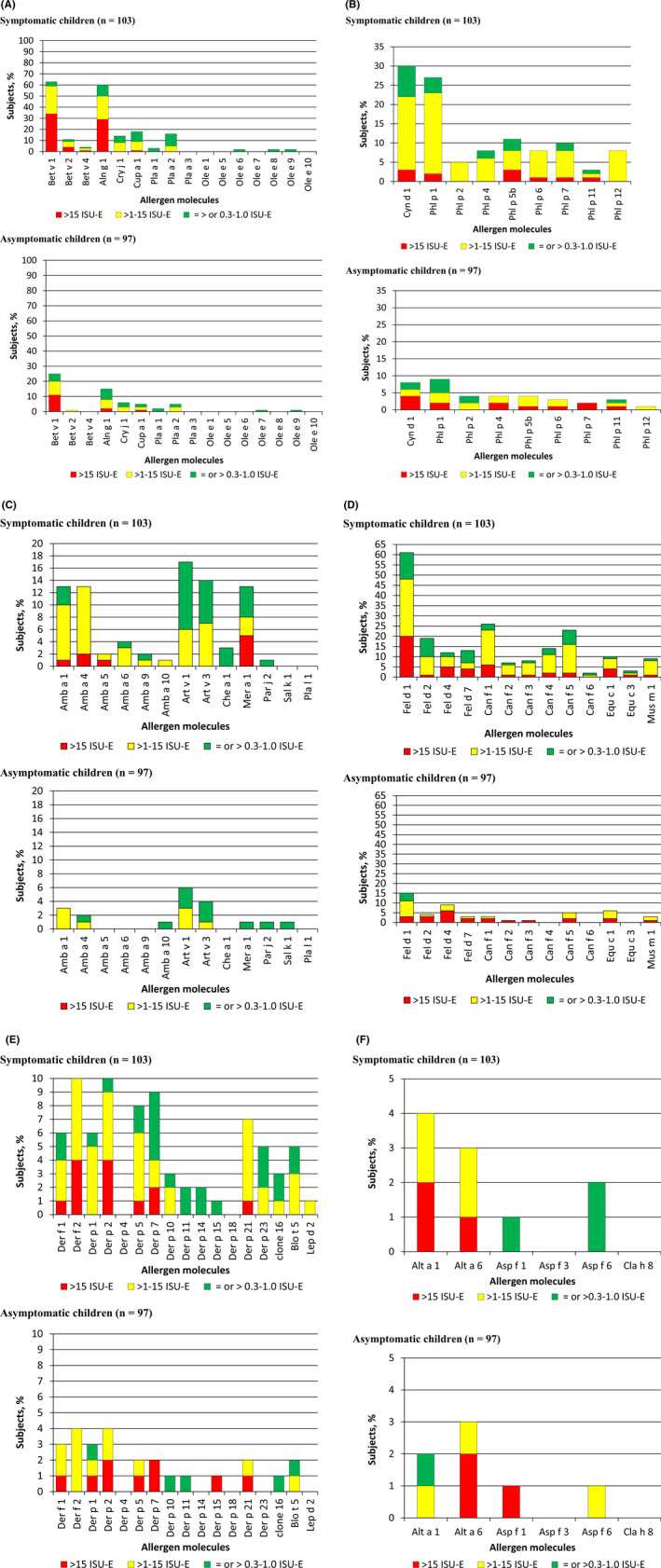
Percentages and levels of specific IgE (*y*‐axes) among children with (upper part) and children without symptoms of allergy (lower part) for tree pollen allergens (A), grass pollen allergens (B), weed pollen allergens (C), animal dander allergens (D), house dust mite allergens (E), and mold allergens (F). Allergen molecules (*x*‐axes) [Colour figure can be viewed at wileyonlinelibrary.com]

Birch pollen was by far the most important allergen source in the Russian population with Bet v 1 being recognized by 63.1% of the symptomatic children. The rates of sensitization to the minor birch pollen allergens Bet v 2 (profilin) and Bet v 4 (calcium‐binding allergen, polcalcin) were 10.6% and 3.9%, respectively, in the symptomatic children (Table S1; Figure 4A). Grass pollen was the next important allergen source with Phl p 1 being the most frequently recognized non‐glycosylated allergen in group 1 children (ie, 27.1%), whereas other grass pollen allergens were much less frequently recognized: Phl p 5 (10.6%) > Phl p 7 (9.7%) > Phl p 4 (7.8%) = Phl p 6 (7.8%) = Phl p 12 (7.8%) > Phl p 2 (4.8%) > Phl p 11 (2.9%) (Table S1; Figure 4B).

After cypress pollen Cup a 1 (18.4%), mugwort pollen was the next important allergen source with Art v 1 and Art v 3 recognized by 16.5% and 13.5% of symptomatic (group 1) children, respectively (Figure 4A,C). Next, in the pollen allergen sources was plane tree with Pla a 2 (15.5%), Pla a 3 (3.9%), and Pla a 1 (2.9%) being recognized by symptomatic children (Figure 4A). Cedar pollen with Cry j 1 (13.5%) and ragweed with Amb a 1 (12.6%), Amb a 4 (9.7%), Amb a 6 (3.9%), Amb a 9 (1.9%), Amb a 5 (0.9%), and Amb a 10 (0.9%) followed then (Figure 4A,C). Interestingly, ash pollen, Parietaria, and saltwort did not play a role as pollen allergen sources.

### 3.6 Indoor allergen sensitization is dominated by cat followed by dog, whereas HDM is less common and has an unusual recognition profile

Besides the major birch pollen allergen, Bet v 1, the major cat allergen Fel d 1 was the most frequently recognized allergen in our population (ie, 61.1% of group 1; 15.4% of group 2) (Table S1; Figure 4D). After Fel d 1, Fel d 2 (19.4%), Fel d 7 (12.6%), and Fel d 4 (11.6%) followed as next frequently recognized cat allergen molecules in group 1 (Table S1; Figure 4D). IgE levels specific for Fel d 7 and Fel d 4 were higher than those for Fel d 2 (Figure 4D; Table S1). The next important indoor allergen source was dog with Can f 1 as the most frequent allergen (26.2%) followed by Can f 5 (23.3%), Can f 4 (13.5%), Can f 3 (7.8%), and Can f 2 (6.8%) among the symptomatic children. Of note, among the animal dander, allergens were the major horse allergen Equ c 1 (group 1:9.7%) and the major mouse allergen, Mus m 1 (group 1:8.7%) (Figure 4D).

Usually, up to 50% of allergic subjects are sensitized to HDM allergen but in our population HDM allergy was quite rare because Der p 2 the most frequently recognized allergen showed IgE reactivity only with 9.7% of children from group 1 (Table S1). Furthermore, the frequencies of IgE recognition of the individual HDM allergen molecules among symptomatic children were unusual because Der p 7 > Der p 5 > Der p 21 were more frequently recognized than Der p 1 and Der f 1 which are usually major HDM allergens for more than 90% of HDM‐allergic patients (Figure 4E).

IgE sensitization to mold allergens was rather rare in our population and the profile was also unusual. For example, Alt a 6 was more often recognized by IgE than the major Alternaria allergen Alt a 1 (Figure 4F).

### 3.7 IgE recognition of other allergen molecules

None of our children showed IgE reactivity to any of the 5 tested latex allergens (Hev b 1, Hev b 3, Hev b 5, Hev b 6, Hev b 8) (Table S3). Also, none of our children displayed IgE reactivity to the bee venom allergens Api m 1, Api m 2, and Api m 4. IgE sensitizations to the major wasp allergen Ves v 5 and Pol d 5 were quite common in asymptomatic children (ie, up to 10.3%) but more rare in symptomatic children (ie, up to 4.8%) (Table S3). No IgE sensitizations against the carbohydrate marker MUXF 3 were found among the symptomatic children, and only one child from the asymptomatic group 2 showed MUXF 3‐specific IgE reactivity (Table S3).

### 3.8 Oligo‐ and polymolecular sensitization profiles dominate among symptomatic children

Figure S1 provides an overview about the percentages of symptomatic (group 1) and asymptomatic (group 2) children with monosensitization, oligosensitization (ie, IgE recognition of 2‐5 non‐cross‐reactive allergens), or polysensitization (>5 allergen molecules). The percentages of monomolecular (14.9%), oligomolecular (20.7%), and polymolecular (14.9%) sensitization profiles were quite comparable in asymptomatic children whereas oligo‐ (30%) and polymolecular sensitization (52.4%) dominated in the symptomatic children over monomolecular sensitization (3.9%) (Figure S1). In 49.5% of the asymptomatic children, no IgE sensitization against any of the allergen on the chip could be detected whereas 13.7% (ie, n = 15) of the symptomatic children were negative at the cutoff level of specific IgE of = or > 0.3 ISU‐IgE. However, 10 out of these 15 children showed allergen‐specific IgE reactivity to at least one molecule >0.1 ISU‐IgE. Thus, only five remained negative (data not shown). As described earlier for adults,^39^ there was no clear association of allergic multimorbidity with oligo‐ or polymolecular IgE sensitization (data not shown).

## 4 DISCUSSION

Our study is the first to perform a meticulous analysis of IgE reactivity profiles to more than 160 allergen molecules in children from Russia (Moscow region) using micro‐arrayed allergen molecules. A unique feature of our study is that we have recruited two age‐ and gender‐matched groups of children, each approximately 100, one with symptoms of allergy and one without symptoms of allergy. For the assignment of children to the symptomatic group 1 and the asymptomatic group 2, we used the internationally standardized ISAAC questionnaire which is established all over the world in different languages.^29^ Our study was designed to include a symptomatic and an asymptomatic group of children to investigate if and how many asymptomatic children show IgE sensitizations to certain allergen molecules and if so, what the differences of molecular IgE sensitizations between the two groups may be. According to the analysis of molecular IgE sensitization profiles with micro‐arrayed allergen molecules in population‐based birth cohort studies, it seems that 50%‐60% of children exhibit IgE sensitizations against at least one of the >160 allergen molecules present on the allergen micro‐array.^24^ In fact, we found that each of the children with symptoms of allergy (ie, 100%) showed IgE reactivity to at least one of the allergen molecules analyzed. Interestingly, 36% of the asymptomatic children also displayed IgE reactivity to at least one of the tested allergen molecules which indicates that more than 30% of persons without symptoms of allergy have a specific IgE sensitization which is in agreement with the results from population‐based birth cohorts. The clinically silent IgE sensitization was also suggested by the positive reactions obtained by skin testing in the asymptomatic children (Table 1).

We know that certain “allergens” have a very low allergenic activity and hence do not induce allergic symptoms in sensitized subjects such as IgE‐reactive carbohydrates.^40^, ^41^

One possible result of the comparison of the molecular IgE sensitization profiles in symptomatic and asymptomatic children could therefore have been that asymptomatic children are primarily sensitized against such low allergenic molecules. Such a result was actually obtained for a population from the Philippines where we detected a high rate of carbohydrate sensitizations in asymptomatic subjects.^28^ However, it is one of the major findings of our study that both symptomatic and asymptomatic children were sensitized against the same allergen molecules. The only difference between the groups was that fewer of the asymptomatic children were sensitized and that allergen‐specific IgE levels were significantly lower among the asymptomatic children. This result underlines the importance of allergen‐specific IgE levels for the development of symptoms. We have not conducted a longitudinal assessment of the asymptomatic children to study if increases of allergen‐specific IgE levels as they occur after frequent allergen contact may be associated with the development of symptoms. However, the longitudinal analysis of children in birth cohorts has shown that with increasing age more children become symptomatic and that this is associated with increases of allergen‐specific IgE levels.^13^ It is therefore conceivable that children from the asymptomatic group may become symptomatic later in life when their allergen‐specific IgE levels increase.

Another major result of our study is that it is the first to establish the molecular IgE sensitization profiles of children in Russia. In this study, we investigated children from the Moscow region in Russia and it must be born in mind that depending on climate and exposomes the IgE sensitization profiles in other regions of Russia may be different from that observed for Moscow. In Moscow, we found a very interesting and peculiar IgE sensitization profile which is characterized by a predominant sensitization to the major birch pollen allergen, Bet v 1,^5^ and the major cat allergen, Fel d 1.^42^ These molecules were recognized by more than 60% of the symptomatic children, and hence, Bet v 1 and Fel d 1 represent the major respiratory molecules. The high rate of sensitization to these two molecules is quite similar to the sensitization profiles observed in Scandinavia and may be due to the high exposure to birch pollen and most likely similar pet‐keeping habits.^13^, ^43^ The high sensitization rate to cat is interesting because only twelve of the children of the symptomatic group had pets at home pointing to the possibility of indirect exposure.

The molecular profiles of respiratory sensitization had a direct influence on the food allergy profiles in Russia (Moscow region) because primary sensitization of birch pollen‐derived Bet v 1 induces cross‐reactive IgE to PR10 food allergens from hazelnut, apple, and other fruits and vegetables which actually dominates food allergy in the Russian children. In fact, almost all food allergic children suffered from oral allergy syndrome (Table 1). This result has important implications for allergen‐specific forms of treatment because allergen‐specific immunotherapy (AIT) with a vaccine conferring cross‐protection to Bet v 1 and the cross‐reactive PR10 food allergens would be needed to treat birch pollen and associated food allergy in our population.^44^ Other forms of food allergy were quite rare. We found that bovine serum albumin, Bos d 6, which may be considered as cow´s milk allergen, was frequently recognized. However, the frequent sensitization to Bos d 6 seems to be due to a primary respiratory sensitization to cat albumin, Fel d 2, because all subjects with Bos d 6‐specific IgE showed IgE reactivity to Fel d 2 and Fel d 2‐specific IgE levels were higher indicating that it was the culprit allergen.

In contrast to Sweden, where genuine peanut allergy which is characterized by sensitization to storage proteins such as Ara h 1, Ara h 2, Ara h 3, and Ara h 6^45^ is common, none of our children showed IgE reactivity to these allergens. Thus, potentially life‐threatening peanut allergy seems to be quite rare in our population which may be due to different feeding/eating habits in Russia where peanuts are not an important part of the diet.

Unlike in Middle Europe where HDM allergy is highly prevalent affecting 30%‐50% of allergic patients,^27^, ^46^ IgE sensitization to HDM allergens was very rare in the Moscow population. This may be due to the climate which is quite similar to Scandinavia (eg, Sweden) where HDM allergy is also rare.^24^ We also found a very interesting IgE sensitization profile to the individual HDM allergens. Unlike in other populations where group 1 HDM allergens (ie, Der p 1, Der f 1) are major allergens recognized by more than 90% of HDM‐allergic patients,^47^ Der p 7, Der p 5, and Der p 21 were more frequently recognized than group 1 HDM allergens. This finding also has therapeutic consequences because it has been shown that commercially available allergen extract‐based HDM allergy vaccines induce mainly group 1‐ and group 2‐specific protective IgG antibodies but fail to protect against Der p 5, Der p 7, Der p 21, and Der p 23.^48^, ^49^ Accordingly, HDM allergy vaccines for our population must be carefully selected to comprise these allergen molecules.

Further characteristics of the investigated children were that grass pollen allergy was dominated by IgE sensitization to group 1 grass pollen allergens and IgE sensitizations to carbohydrate epitopes as detected by CCD molecules on the chip were rare in our population. The IgE sensitization rate to natural Phl p 4 which may be considered a marker for CCD sensitization was much lower in our population than for example in children from Sweden^50^ but we noted frequent IgE sensitizations to natural glycosylated allergen molecules from Bermuda grass (Cyn d 1), cedar (Cry j 1), and cypress (Cup a 1). Whether these IgE sensitizations are directed against the carbohydrate or protein moieties of these allergens cannot be firmly established at present. The relatively high frequency of sensitization to cypress in children in Moscow was a surprise. There are several possible explanations for this observation. nCup a 1 is a glycosylated allergen, and hence, cross‐reactivity with other carbohydrate epitope‐bearing allergens is possible. Second, although cypress trees are not really found in the Moscow region, some people use them as decorative plants indoors and they are also frequently used as a part of landscape design in public places. Third, pediatricians recommend avoiding domestic allergens and traveling to the southern regions. Accordingly, allergic children from Moscow may spend 1‐2 months in the south every year where cypress is common.

Our study has some limitations because we have only analyzed a relatively limited number of children from the Moscow region and it is quite possible that children from other regions of Russia may show different IgE sensitization profiles due to different climate and exposomes. Hence, larger studies involving different regions of Russia will be necessary to establish a complete molecular allergen map for the complete country. The children analyzed in our study were approximately 12 years old, and we therefore think that they have already acquired most of their IgE sensitizations. Studies of birth cohorts indeed indicate that there are no big changes in IgE reactivity profiles between this age and adolescence.^24^

In summary, our study is the first cross‐sectional study to compare molecular IgE sensitization profiles to a large number of allergen molecules in symptomatic and asymptomatic children in an as yet not investigated part of the world. The study design employed by us will be useful also for other populations and areas in the world. The obtained results have important implications for allergen‐specific forms of treatment and prevention which can be implemented in regional allergy treatment and prevention programs especially in childhood.

## CONFLICT OF INTEREST

RV has received grants from the Austrian Science Fund (FWF), Viravaxx, Vienna, Austria, and HVD Life Sciences, Vienna, Austria, and is recipient of a Megagrant of the Government of the Russian Federation, grant number 14.W03.31.0024. He serves as a consultant for Viravaxx, Vienna, Austria. Christian Lupinek has received honoraria from Thermo Fisher for lectures. All other authors declare that they have no relevant conflict of interest.

## AUTHOR CONTRIBUTION

**Olga Gurevna Elisyutina:** Data curation (equal); Formal analysis (lead); Methodology (lead); Validation (lead); Visualization (lead); Writing‐original draft (equal); Writing‐review & editing (equal). **Christian Lupinek:** Data curation (equal); Formal analysis (equal); Methodology (equal); Software (equal); Validation (equal); Visualization (equal); Writing‐review & editing (equal). **Elena Sergeevna Fedenko:** Data curation (lead); Formal analysis (equal); Methodology (equal); Project administration (equal); Supervision (equal); Validation (equal); Writing‐review & editing (equal). **Alla Olegovna Litovkina:** Data curation (supporting); Formal analysis (supporting); Resources (equal); Validation (equal); Writing‐review & editing (equal). **Evgenii Valentinovich Smolnikov:** Data curation (supporting); Formal analysis (supporting); Resources (equal); Software (supporting); Validation (supporting); Visualization (equal); Writing‐review & editing (equal). **Nataliya Ivanovna Ilina:** Data curation (equal); Formal analysis (equal); Methodology (equal); Supervision (lead); Validation (supporting); Writing‐review & editing (equal). **Dmitry Anatolevich Kudlay:** Data curation (supporting); Formal analysis (equal); Methodology (equal); Supervision (equal); Writing‐review & editing (equal). **Igor Petrovich Shilovskiy:** Data curation (equal); Formal analysis (equal); Methodology (supporting); Supervision (equal); Validation (equal); Writing‐review & editing (equal). **Rudolf Valenta:** Conceptualization (lead); Formal analysis (lead); Funding acquisition (lead); Project administration (lead); Supervision (equal); Validation (equal); Visualization (equal); Writing‐original draft (lead); Writing‐review & editing (lead). **Musa Khaitov:** Conceptualization (lead); Investigation (lead); Project administration (lead); Supervision (lead).

### Peer Review

The peer review history for this article is available at https://publons.com/publon/10.1111/pai.13354.

## Supporting information

Fig S1

Table S1

Table S2

Table S3
