# Supplementary figures and images for: IgE‐reactivity profiles to allergen molecules in Russian children with and without symptoms of allergy revealed by micro‐array analysis
Source: Pediatr Allergy Immunol. 2020 Oct 4;32(2):251–63. doi: 10.1111/pai.13354 (PMC7891667; doi:10.1111/pai.13354)

Figure E1

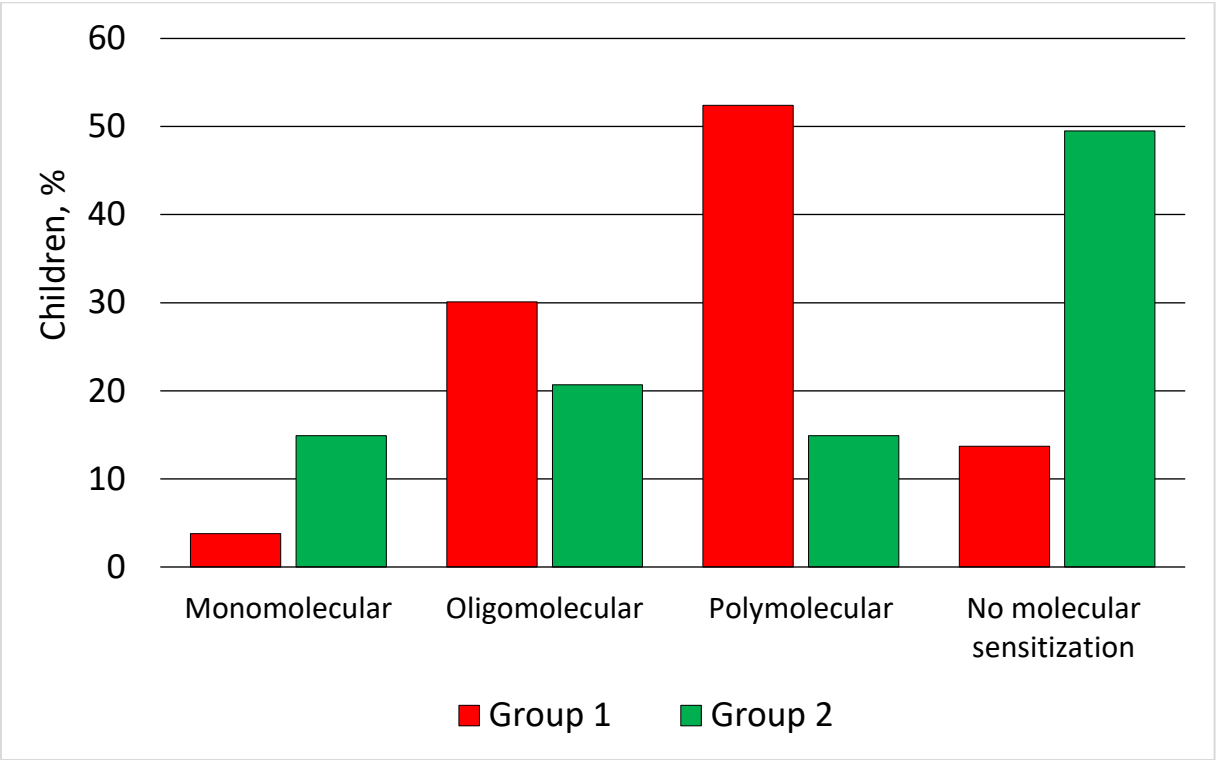

Supplement: Supplementary file 1 — Fig S1 [file PAI-32-251-s001.pdf]
