# Supplementary material for: IgE‐reactivity profiles to allergen molecules in Russian children with and without symptoms of allergy revealed by micro‐array analysis
Source: Pediatr Allergy Immunol. 2020 Oct 4;32(2):251–63. doi: 10.1111/pai.13354 (PMC7891667; doi:10.1111/pai.13354)
Supplement: Supplementary file 2 — Table S1 [file PAI-32-251-s002.docx]

**Table S1.**

| **Allergens** | **Group 1**  **(Patients with symptoms of allergy), n=103** | | **Group 2**  **(Subjects without symptoms of allergy), n=97** | | **Specific IgE group 1 vs. group 2**  **p values** |
| --- | --- | --- | --- | --- | --- |
|  | IgE, ISU-E,  Me [Q_1;_ Q_3_] | Numbers of positive subjects,  n (%) | IgE, ISU-E,  Me [Q_1_; Q_3_] | Numbers of positive subjects,  n (%) |  |
| rBet v 1 | 18.7  [3.27; 57.2] | 65 (63.1) | 1.12  [0.44; 6.37] | 25 (25.7) | **p<0.0001** |
| rFel d 1 | 6.45  [1.5; 32.44] | 63 (61.1) | 3.71  [1.52; 19.89] | 15 (15.4) | **p<0.0001** |
| rAln g 1 | 13.83  [3.16; 44.2] | 62 (60.2) | 3.58  [1.34; 24.07] | 15 (15.4) | **p<0.0001** |
| nCyn d 1 | 3.16  [0.85; 6.45] | 31 (30.1) | 0.91  [0.83; 19.85] | 7 (7.2) | **p<0.001** |
| rPhl p 1 | 3.82  [1.69; 8.37] | 28 (27.1) | 7.98  [0.45; 13.25] | 9 (9.2) | **p<0.001** |
| rCan f 1 | 4.13  [2.07; 14.47] | 27 (26.2) | 0.82  [0.3; 1.6] | 3 (3.1) | **p<0.001** |
| rCan f 5 | 2.19  [0.67; 4.06] | 24 (23.3) | 2.33  [0.73; 4.07] | 5 (5.1) | **p<0.0001** |
| nFel d 2 | 1.09  [0.65; 3.81] | 20 (19.4) | 0.55  [0.46; 1.03] | 4 (4.1) | **p<0.001** |
| nCup a 1 | 0.98  [0.55; 2.06] | 19 (18.4) | 1.68  [0.87; 1.78] | 5 (5.1) | **p<0.001** |
| nArt v 1 | 0.78  [0.41; 1.04] | 17 (16.5) | 0.36  [0.34; 0.51] | 6 (6.2) | **p<0.001** |
| rPla a 2 | 0.59  [0.41; 1.81] | 16 (15.5) | 0.85  [0.47; 1.69] | 5 (5.1) | **p<0.05** |
| rCan f 4 | 2.34  [0.79; 2.36] | 14 (13.5) | 0 | 0 | n.a. |
| nCry j 1 | 1.30  [0.7; 3.37] | 14 (13.5) | 1.28  [0.73; 1.69] | 6 (6.2) | 0.0696 |
| nArt v 3 | 0.93  [0.77; 3.37] | 14 (13.5) | 0.52  [0.42; 0.74] | 4 (4.1) | **p<0.001** |
| rMer a 1 | 9.33  [0.51; 25.8] | 13 (12.6) | 7.77  [7.77; 7.77] | 1 (1.03) | **p<0.001** |
| rFel d 7 | 1.05  [0.66; 30.65] | 13 (12.6) | 0.51  [0.44; 0.57] | 3 (3.1) | **p<0.001** |
| nAmb a 1 | 1.14  [0.21; 3.07] | 13 (12.6) | 3.07  [2.1; 3.75] | 3 (3.1) | **p<0.001** |
| rFel d 4 | 6.85  [1.2; 5.89] | 12 (11.6) | 4.34  [0.83; 7.27] | 9 (9.2) | **p<0.001** |
| rBet v 2 | 11.29  [1.04; 24.09] | 11 (10.6) | 4.54  [4.54; 4.54] | 1 (1.03) | **p<0.001** |
| rPhl p 5b | 12.64  [4.23; 37.56] | 11 (10.6) | 43.88  [4.82; 101.45] | 4 (4.1) | 0.0783 |
| rAmb a 4 | 1.19  [1.02; 2.00] | 10 (9.7) | 2.87  [0.55; 5.19] | 2 (2.1) | **p<0.05** |
| rDer f 2 | 14.31  [12.74;25.37] | 10 (9.7) | 20.43  [2.79; 42.37] | 4 (4.1) | 0.0614 |
| rDer p 2 | 13.61  [11.4; 24.08] | 10 (9.7) | 17.72  [2.73; 36.78] | 4 (4.1) | **p<0.05** |
| rEqu c 1 | 2.04  [0.8; 25.31] | 10 (9.7) | 0.62  [0.54; 8.0] | 6 (6.2) | 0.1476 |
| rPhl p 7 | 2.56  [1.17; 4.39] | 10 (9.7) | 7.72  [7.68; 7.75] | 2 (2.1) | **p<0.01** |
| rDer p 7 | 2.10  [1.9; 2.67] | 9 (8.7) | 0.92  [0.83; 1.0] | 2 (2.1) | 0.0648 |
| nMus m 1 | 0.93  [0.36; 6.49] | 9 (8.7) | 0.62  [0.48; 1.16] | 3 (3.1) | 0.0914 |
| rAlt a 6 | 1.83  [0.44; 14.75] | 8 (7.8) | 0.77  [0.63; 1.25] | 3 (3.1) | **p<0.05** |
| rDer p 5 | 4.76  [3.16; 7.46] | 8 (7.8) | 2.99  [0.94; 5.04] | 2 (2.1) | **p<0.05** |
| nCan f 3 | 1.84  [0.42; 4.36] | 8 (7.8) | 0.57  [0.57; 0.57] | 1 (1.03) | **p<0.05** |
| nPhl p 4 | 1.87  [0.69; 5.09] | 8 (7.8) | 0.89  [0.6; 7.71] | 4 (4.1) | 0.1908 |
| rPhl p 6 | 3.37  [1.49; 5.16] | 8 (7.8) | 15.6  [0.53; 34.75] | 3 (3.1) | 0.2390 |
| rPhl p 12 | 3.68  [1.35; 10.86] | 8 (7.8) | 1.96  [1.96; 1.96] | 1 (1.03) | **p<0.01** |
| rDer p 21 | 3.10  [2.14; 11.47] | 7 (6.8) | 5.96  [5.28; 6.65] | 2 (2.1) | 0.0909 |
| rCan f 2 | 2.15  [0.35; 4.52] | 7 (6.8) | 0 | 0 | n.a. |
| nDer f 1 | 8.49  [0.88; 14.78] | 6 (5.8) | 3.07  [0.49; 27.21] | 3 (3.1) | 0.1956 |
| nDer p 1 | 8.50  [3.43; 12.51] | 6 (5.8) | 2.12  [0.41; 21.98] | 3 (3.1) | 0.1956 |
| rBlo t 5 | 1.91  [1.77; 3.71] | 5 (4.8) | 3.38  [0.95; 4.81] | 2 (2.1) | 0.4761 |
| rDer p 23 | 0.79  [0.69; 4.66] | 5 (4.8) | 0 | 0 | n.a. |
| rPhl p 2 | 5.28  [3.07; 5.99] | 5 (4.8) | 1.11  [0.66; 2.09] | 4 (4.1) | 0.9741 |
| rAlt a 1 | 19.81  [13.4; 24.36] | 4 (3.9) | 0.37  [0.37; 0.37] | 1 (1.03) | 0.0871 |
| rBet v 4 | 4.30  [1.04; 56.17] | 4 (3.9) | 0 | 0 | n.a. |
| rAmb a 6 | 0.73  [0.64; 2.26] | 4 (3.9) | 0 | 0 | n.a. |
| rPla a 3 | 0.91  [0.45; 1.17] | 4 (3.9) | 0.7  [0.7; 0.7] | 1 (1.03) | 0.876 |
| rDer p 10 | 1.28  [0.97; 1.43] | 3 (2.9) | 34.54  [34.54; 34.54] | 1 (1.03) | 0.182 |
| rChe a 1 | 0.72  [0.4; 0.91] | 3 (2.9) | 0 | 0 | n.a. |
| nEqu c 3 | 4.39  [0.53; 20.49] | 3 (2.9) | 0 | 0 | n.a |
| rPla a 1 | 0.47  [0.43; 0.76] | 3 (2.9) | 0.41  [0.37; 0.45] | 2 (2.1) | 0.6417 |
| rPhl p 11 | 7.63  [0.93; 8.31] | 3 (2.9) | 0.73  [0.62; 58.42] | 3 (3.1) | 0.9934 |
| rclone 16 | 0.47  [0.38; 8.88] | 3 (2.9) | 1.02  [1.02; 1.02] | 1 (1.03) | 0.5656 |
| rAmb a 9 | 4.30  [0.42; 83.2] | 2 (1.9) | 0 | 0 | n.a. |
| rAsp f 6 | 6.04  [0.64; 11.45] | 2 (1.9) | 1.93  [1.93; 1.93] | 1 (1.03) | 0.5735 |
| rDer p 11 | 0.40  [0.4; 0.41] | 2 (1.9) | 1.78  [1.78; 1.78] | 1 (1.03) | 0.6417 |
| rDer p 14 | 0.54  [0.39; 0.69] | 2 (1.9) | 0 | 0 | n.a. |
| rCan f 6 | 1.80  [0.84; 2.77] | 2 (1.9) | 0 | 0 | n.a. |
| rOle e 6 | 13.44  [2.83; 24.06] | 2 (1.9) | 0 | 0 | n.a. |
| rOle e 8 | 16.88  [2.89; 30.88] | 2 (1.9) | 0 | 0 | n.a. |
| rAmb 5 | 0.83  [0.83; 0.83] | 1 (0.9) | 0 | 0 | n.a. |
| rAsp f 1 | 0.48  [0.48; 0.48] | 1 (0.9) | 0 | 0 | n.a. |
| rBla g 1 | 0.72  [0.72; 0.72] | 1 (0.9) | 0 | 0 | n.a. |
| rBla g 2 | 0.41  [0.41; 0.41] | 1 (0.9) | 0 | 0 | n.a. |
| rBla g 5 | 45.6  [45.6; 45.6] | 1 (0.9) | 6.59  [6.59; 6.59] | 1 (1.03) | 0.9959 |
| nBla g 7 | 0.71  [0.71; 0.71] | 1 (0.9) | 31.22  [31.22;31.22] | 1 (1.03) | 1.0000 |
| rDer p 15 | 0.60  [0.60; 0.60] | 1 (0.9) | 0.72  [0.72; 0.72] | 1(1.03) | 1.0000 |
| rLep d 2 | 3.56  [3.56; 3.56] | 1(0.9) | 0 | 0 | n.a. |
| rOle e 9 | 1.41  [1.41; 1.41] | 1 (0.9) | 0.36  [0.36; 0.36] | 1 (1.03) | 0.5158 |
| rPar j 2 | 0.7  [0.7; 0.7] | 1 (0.9) | 0 | 0 | n.a. |
| rAmb a 10 | 7.46  [7.46;7.46] | 1 (0.9) | 0 | 0 | n.a. |
| rAsp f 3 | 0 | 0 | 0 | 0 | n.a. |
| rCla h 8 | 0 | 0 | 0 | 0 | n.a. |
| rDer p 18 | 0 | 0 | 0 | 0 | n.a. |
| rDer p 4 | 0 | 0 | 0 | 0 | n.a. |
| rOle e 1 | 0 | 0 | 0 | 0 | n.a. |
| nOle e 7 | 0 | 0 | 0.59  [0.59; 0.59] | 1 (1.03) | n.a. |
| rOle e 5 | 0 | 0 | 0 | 0 | n.a. |
| rOle 10 | 0 | 0 | 0 | 0 | n.a. |
| rPla l 1 | 0 | 0 | 0 | 0 | n.a. |
| nSal k 1 | 0 | 0 | 0.38  [0.38; 0.38] | 1 (1.03) | n.a. |
| rPar j 1 | 0 | 0 | 0.53  [0.53; 0.53] | 1 (1.03) | n.a. |
| rTri a Trx | 0 | 0 | 0 | 0 | n.a. |
| rTri a 39 (10) | 0 | 0 | 0 | 0 | n.a. |
| rTri a GST | 0 | 0 | 0 | 0 | n.a. |
| rTri a 32 | 0 | 0 | 0 | 0 | n.a. |
| rTri a 12 | 0 | 0 | 0 | 0 | n.a. |
| rTri a DH | 0 | 0 | 0 | 0 | n.a. |
| rTri a 32 | 0 | 0 | 0 | 0 | n.a. |
