# Supplementary material for: IgE‐reactivity profiles to allergen molecules in Russian children with and without symptoms of allergy revealed by micro‐array analysis
Source: Pediatr Allergy Immunol. 2020 Oct 4;32(2):251–63. doi: 10.1111/pai.13354 (PMC7891667; doi:10.1111/pai.13354)
Supplement: Supplementary file 3 — Table S2 [file PAI-32-251-s003.docx]

**Table S2.**

| **Allergens** | **Group 1**  **(Patients with symptoms of allergy), n=103** | | **Group 2**  **(Subjects without symptoms of allergy), n=97** | | **Specific IgE group 1 vs. group 2**  **p values** |
| --- | --- | --- | --- | --- | --- |
|  | IgE, ISU-E,  Me [Q_1;_ Q_3_] | Numbers of positive subjects,  n (%) | IgE, ISU-E,  Me [Q_1;_ Q_3_] | Numbers of positive subjects,  n (%) |  |
| rCor a 1.0401 | 6.19  [2.46; 21.13] | 54 (52.4) | 1.77  [0.51; 14.31] | 14 (14.4) | **p<0.0001** |
| rMal d 1 | 7.48  [1.49; 24.71] | 53 (51.4) | 1.03  [0.66; 4.13] | 12 (12.3) | **p<0.0001** |
| rAra h 8 | 4.84  [1.53; 17.76] | 47 (45.6) | 1.44  [0.36; 5.46] | 9 (9.2) | **p<0.0001** |
| rPru p 1 | 3.61  [1.22; 16.1] | 44 (42.7) | 0.90  [0.64; 7.68] | 10 (10.3) | **p<0.0001** |
| rGly m 4 | 3.29  [1.21; 17.6] | 42 (40.7) | 0.72  [0.5; 4.87] | 10 (10.3) | **p<0.0001** |
| rApi g 1 | 2.88  [1.22; 11.33] | 36 (34.9) | 2.55  [1.01; 4.2] | 4 (4.1) | **p<0.001** |
| rAct d 8 | 2.42  [0.67; 5.28] | 25 (24.3) | 2.66  [2.31; 3.02] | 4 (4.1) | **p<0.0001** |
| nBos d 6 | 0.53  [0.37; 1.07] | 17 (16.5) | 0.36  [0.34; 0.66] | 5 (5.1) | **p<0.001** |
| nJug r 2 | 0.67  [0.5; 1.55] | 15 (14.5) | 1.36  [1.17; 1.55] | 4 (4.1) | **p<0.001** |
| rGad c 1 | 1.17  [0.56; 3.56] | 8 (7.8) | 0.31  [0.31; 0.31] | 1 (1.03) | **p<0.001** |
| rJug r 3 | 0.48  [0.38; 0.93] | 8 (7.8) | 1.13  [1.13; 1.13] | 1 (1.03) | **p<0.05** |
| nAct d 1 | 4.63  [1.62; 19.85] | 7 (6.8) | 1.11  [1.11; 1.11] | 1 (1.03) | **p<0.05** |
| rAra h 9 | 0.45  [0.36; 1.49] | 6 (5.8) | 0.87  [0.64; 1.15] | 3 (3.1) | 0.2339 |
| rCor a 8 | 0.47  [0.38; 0.48] | 5 (4.8) | 0.70  [0.37; 1.03] | 2 (2.1) | **p<0.05** |
| nGal d 3 | 7.06  [1.85; 28.95] | 4 (3.9) | 0 | 0 | n.a. |
| nAct d 2 | 0.43  [0.37; 0.59] | 4 (3.9) | 1.88  [1.88; 1.88] | 1 (1.03) | 0.2480 |
| rPru p 3 | 0.69  [0.4; 0.97] | 3 (2.9) | 0.57  [0.57; 0.57] | 1 (1.03) | 0.2932 |
| nGly m 5 | 0.44  [0.39; 0.69] | 3 (2.9) | 0.42  [0.42; 0.42] | 1 (1.03) | 0.4541 |
| nAna o 2 | 0.64  [0.3; 0.98] | 2 (1.9) | 0 | 0 | n.a. |
| nGal d 2 | 1.98  [1.57; 2.39] | 2 (1.9) | 0 | 0 | n.a |
| nCor a 9 | 0.70  [0.57; 0.83] | 2 (1.9) | 0.32  [0.32; 0.32] | 1 (1.03) | 0.4059 |
| nPen m 4 | 0.77  [0.62; 0.92] | 2 (1.9) | 0 | 0 | n.a. |
| rJug r 1 | 2.55  [0.62; 23.4] | 2 (1.9) | 0.62  [0.62; 0.62] | 1 (1.03) | 0.5578 |
| nGal d 1 | 0.78  [0.78; 0.78] | 1 (0.9) | 0 | 0 | n.a. |
| nGal d 5 | 0.56  [0.56; 0.56] | 1 (0.9) | 0 | 0 | n.a. |
| rAna o 3 | 1.81  [1.81; 1.81] | 1 (0.9) | 0 | 0 | n.a. |
| rAna o 1 | 0.38  [0.38; 0.38] | 1 (0.9) | 0.50  [0.50; 0.50] | 1 (1.03) | n.a. |
| nBos d Lactoferrin | 2.5  [2.5; 2.5] | 1 (0.9) | 0 | 0 | n.a. |
| nAra h 1 | 1.15  [1.15; 1.15] | 1 (0.9) | 0 | 0 | n.a. |
| rPis v 3 | 0.45  [0.45; 0.45] | 1 (0.9) | 0.99   [0.88; 1.1] | 2 (2.1) | 0.5578 |
| nPen m 2 | 0.36  [0.36; 0.36] | 1 (0.9) | 3.02  [1.34; 3.45] | 3 (3.1) | 0.3967 |
| nPen m 1 | 1.06  [1.06; 1.06] | 1 (0.9) | 20.6  [0.57; 40.62] | 2 (2.1) | 0.3185 |
| nSes i 1 | 0.99  [0.99; 0.99] | 1 (0.9) | 0 | 0 | n.a. |
| m82 | 0.33  [0.33; 0.33] | 1 (0.9) | 0 | 0 | n.a. |
| rCor a 14 | 0 | 0 | 0 | 0 | n.a. |
| nAna c 2.01 | 0 | 0 | 1.48  [1.48; 1.48] | 1 (1.03) | n.a. |
| rBer e 1 | 0 | 0 | 0 | 0 | n.a. |
| nFag e 2 | 0 | 0 | 0 | 0 | n.a. |
| Alpha S1 casein 1 | 0 | 0 | 0.4  [0.4; 0.4] | 1 (1.03) | n.a. |
| nBos d 4 | 0 | 0 | 0 | 0 | n.a. |
| nBos d 5 | 0 | 0 | 0 | 0 | n.a. |
| nBos d 8 | 0 | 0 | 0 | 0 | n.a. |
| rBos d 9 | 0 | 0 | 0 | 0 | n.a. |
| rBos d 10 | 0 | 0 | 0 | 0 | n.a. |
| rBos d 11 | 0 | 0 | 0 | 0 | n.a. |
| rBos d 12 | 0 | 0 | 0 | 0 | n.a. |
| aS1 | 0 | 0 | 0 | 0 | n.a. |
| aS2 | 0 | 0 | 0 | 0 | n.a. |
| Alpha S2 casein | 0 | 0 | 0 | 0 | n.a. |
| rPru du 3 | 0 | 0 | 0 | 0 | n.a. |
| rPru du 4 | 0 | 0 | 0 | 0 | n.a. |
| rPru du 6 | 0 | 0 | 0 | 0 | n.a. |
| rPru du 6.01 | 0 | 0 | 0 | 0 | n.a. |
| rPru du 6.02 | 0 | 0 | 0 | 0 | n.a. |
| nAra h 3 | 0 | 0 | 0 | 0 | n.a. |
| nAra h 6 | 0 | 0 | 0 | 0 | n.a. |
| rAra h 2 | 0 | 0 | 0 | 0 | n.a. |
| nGly m 6 | 0 | 0 | 0 | 0 | n.a. |
| nTri a aA_TI | 0 | 0 | 0.83  [0.83; 0.83] | 1 (1.03) | n.a. |
| rTri a 19.0101 | 0 | 0 | 0 | 0 | n.a. |
| rTri a 14 | 0 | 0 | 0.43  [0.43; 0.43] | 1 (1.03) | n.a |
| rTri a 36(191_369) | 0 | 0 | 0 | 0 | n.a. |
| rTri a 36 | 0 | 0 | 0 | 0 | n.a. |
| rm 43 | 0 | 0 | 0 | 0 | n.a. |
| rTri a GG1 | 0 | 0 | 0 | 0 | n.a. |
| rPeptide 4 | 0 | 0 | 0 | 0 | n.a. |
| rPeptide 18 | 0 | 0 | 0 | 0 | n.a. |
| rClone 79 | 0 | 0 | 0 | 0 | n.a. |
| rClone 85 | 0 | 0 | 0 | 0 | n.a. |
| rClone 110 | 0 | 0 | 0 | 0 | n.a. |
| rTri a bA | 0 | 0 | 0 | 0 | n.a. |
| rAvenin-like protein | 0 | 0 | 0 | 0 | n.a. |
