# Supplementary material for: IgE‐reactivity profiles to allergen molecules in Russian children with and without symptoms of allergy revealed by micro‐array analysis
Source: Pediatr Allergy Immunol. 2020 Oct 4;32(2):251–63. doi: 10.1111/pai.13354 (PMC7891667; doi:10.1111/pai.13354)
Supplement: Supplementary file 4 — Table S3 [file PAI-32-251-s004.docx]

**Table S3.**

| **Allergens** | **Group 1**  **(Patients with symptoms of allergy), n=103** | | **Group 2 (Subjects without symptoms of allergy), n=97** | | **Specific IgE group 1 vs. group 2**  **p value** |
| --- | --- | --- | --- | --- | --- |
|  | IgE, ISU-E,  Me [Q_1;_ Q_3_] | Numbers of positive subjects,  n (%) | IgE, ISU-E,  Me [Q_1;_ Q_3_] | Numbers of positive subjects,  n (%) |  |
| CCD | 0.7  [0.56; 2.35] | 11 | 0 | 0 | n.a. |
| rPol d 5 | 0.66  [0.54; 0.91] | 5 (4.8) | 0.90  [0.43; 1.39] | 7 (7.2) | 0.5783 |
| rVes v 5 | 0.94  [0.6; 1.27] | 2 (1.9) | 0.72  [0.72; 0.72] | 1 (1.03) | 0.6821 |
| rAni s 3 | 1.11  [0.71; 1.5] | 2 (1.9) | 62.7  [62.7; 62.7] | 1 (1.03) | 0.5735 |
| 2NMyo | 0.71  [0.71; 0.71] | 1 (0.9) | 0 | 0 | n.a. |
| MUXF3 | 1.48  [1.48; 1.48] | 1 (0.9) | 0 | 0 | n.a. |
| NMyo | 0 | 0 | 0 | 0 | n.a. |
| Alpha Gal | 0 | 0 | 0 | 0 | n.a. |
| rAni s 1 | 0 | 0 | 0 | 0 | n.a. |
| rVes v 1 | 0 | 0 | 0 | 0 | n.a. |
| rApi m 1 | 0 | 0 | 0 | 0 | n.a. |
| nApi m 2 | 0 | 0 | 0 | 0 | n.a. |
| nApi m 4 | 0 | 0 | 0 | 0 | n.a. |
| rHev b 5 | 0 | 0 | 0 | 0 | n.a. |
| rHev b 3 | 0 | 0 | 0 | 0 | n.a. |
| rHev b 6.01 | 0 | 0 | 0 | 0 | n.a. |
| rHev b 1 | 0 | 0 | 0 | 0 | n.a. |
| rHev b 8 | 0 | 0 | 0 | 0 | n.a. |
| rhtTG2 | 0 | 0 | 0 | 0 | n.a. |
